# Smart Sensing Multifunctionalities Based on Barium Strontium Titanate Thin Films

**DOI:** 10.3390/s22197183

**Published:** 2022-09-22

**Authors:** Linghua Wang, Minmin Zhu, Yong Shao, Yida Zhao, Can Wei, Langfeng Gao, Yiping Bao

**Affiliations:** 1 College of Physics and Information Engineering, Fuzhou University, Fuzhou 350108, China; 2 FZU-Jinjiang Joint Institute of Microelectronics, Jinjiang Science and Education Park, Fuzhou University, Jinjiang 362200, China; 3 School of Materials Science and Engineering, Nanyang Technological University, 50 Nanyang Avenue, Singapore 639798, Singapore; 4 Academy of Hi-Tech Research, Hunan Institute of Traffic Engineering, Hengyang 421099, China

**Keywords:** barium strontium titanate, sensor, force, optical manipulation, Pockels effect

## Abstract

Sensors that have low power consumption, high scalability and the ability of rapidly detecting multitudinous external stimulus are of great value in cyber-physical interactive applications. Herein, we reported the fabrication of ferroelectric barium strontium titanate ((Ba_70_Sr_30_)TiO_3_, BST) thin films on silicon substrates by magnetron sputtering. The as-grown BST films have a pure perovskite structure and exhibit excellent ferroelectric characteristics, such as a remnant polarization of 2.4 μC/cm^2^, a ferro-to-paraelectric (tetragonal-to-cubic) phase transition temperature of 31.2 °C, and a broad optical bandgap of 3.58 eV. Capacitor-based sensors made from the BST films have shown an outstanding average sensitivity of 0.10 mV·Pa^−1^ in the 10–80 kPa regime and work extremely steadily over 1000 cycles. More importantly, utilizing the Pockels effect, optical manipulation in BST can be also realized by a smaller bias and its electro-optic coefficient r_eff_ is estimated to be 83.5 pmV^−1^, which is 2.6 times larger than in the current standard material (LiNbO_3_) for electro-optical devices. Our work established BST thin film as a powerful design paradigm toward on-chip integrations with diverse electronics into sensors via CMOS-comparable technique.

## 1. Introduction

With the rapid development of the Internet of Things (IoT), a gigantic array of electronics and sensors have been quietly functioning in our daily life, allowing for signal processing, wireless communication, edge computing and many more [1,2,3]. In particular, the sensors are the core components that directly enable interconversion between the external stimuli and the signals. In recent years, great efforts have been dedicated to achieving highly selective and highly scalable sensors. Among them, both capacitive- and transistor-based sensors have attracted considerable interest, owing to their simple designs, lower power consumption, and complementary metal oxide semiconductor (CMOS)-comparable manufacture technique [4,5,6,7]. They can be integrated with a diverse range of electronics into sensors for monitoring optical, mechanical, thermal and vital parameters, thereby demonstrating the great potential of numerous existing and emerging applications, such as human–machine interfaces, robotic technologies, and wearable healthcare monitors. 

Noticeably, lead-free ferroelectric materials have emerged as a viable platform for multimodal distributed sensing functionalities, owing to their high capacitance, switchable spontaneous polarization, pyroelectric, piezoelectric, photovoltaic, and electro-optic effects [3,8,9,10,11]. The unique characteristics of ferroelectrics can be utilized to measure pressure, force, temperature, humidity, and IR radiation [12,13]. Tian et al. investigated reversible bulk polarization switching induced by construction of chemical bonds at the surface of ferroelectric BiFeO_3_ in aqueous solution, opening up new opportunities for chemical stimuli sensing [14]. Additionally, Yang group designed a vertical ITO/BaTiO_3_/ITO photodetector, which generates a dramatic increase of photocurrent, revealing the potential for optical monitoring [15]. However, aforementioned works either adopt bulk ferroelectric materials or single crystal substrate, such as SrTiO_3_, which cannot simultaneously achieve both lower power consumption and CMOS-comparable technology. Currently, the fabrication of ferroelectric thin films directly grown on Si wafer remains challenging, because most perovskite materials, such as (Pb,Zr)TiO_3_, BiFeO_3_ and Pb(Mg_2/3_Nb_1/3_)O_3_-PbTiO_3_, require a certain degree of lattice matching with the substrate to obtain a pure perovskite phase [14,16,17]. In this letter, we reported the growth of high-quality ferroelectric BST thin films on Si substrates by magnetron sputtering. Subsequently, the formation mechanism of perovskite phase, spontaneous polarization and temperature-dependent dielectric characteristics were investigated. Additionally, capacitor-based BST sensors have exhibited a remarkable sensitivity of 0.10 mV·Pa^−1^ in the range from 10 kPa to 80 kPa, and even work extremely steadily on over 1000 cycles. Optical performances of BST thin films can be easily manipulated by a smaller external voltage, indicating the great potential for the current standard material (LiNbO_3_) replacement. Owing to their excellent ferroelectric properties, mechanical and optical manipulation in the resulting BST films were realized, providing a new opportunity for multifunctional sensing applications.

## 2. Experimental Section

BST thin films were synthesized at a substrate temperature of around 300 °C and at a mixed Ar/O_2_ gas atmosphere by magnetron sputtering, followed by a post-annealing process at 680 °C for 10 min. During the deposition, the pressure is about 1 Torr and the power is 150 W. The resulting BST thin film is calculated to be 500 nm. X-ray diffraction (XRD, 20 kV) with Cu kα anode and scanning electron microscopy (SEM, 5 kV) equipped with energy-dispersive X-ray spectroscopy was carried out to investigate the crystallinity and the component of the as-grown BST thin films. The polarization–electric field (P-E) loop and temperature-dependent dielectric characteristics were measured by a high-precision LCR meter and Radiant precision multiferroic II. As for the mechanical manipulation, one setup was used to record the output voltage generated by BST films under the various pressures. Transmittance spectrum and the refractive index of all samples were characterized using a UV–Vis–NIR spectrometer and an ellipsometry. Lastly, a customer-designed facility equipped with a laser, two polarizers, an analyzer, a photodetector and a power amplifier were used to realize the optical manipulation.

## 3. Results and Discussion

**Figure 1:**
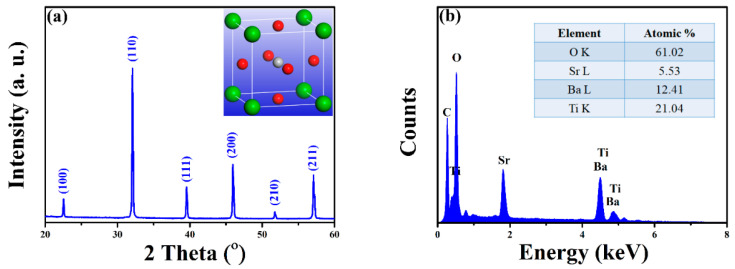
(**a**) XRD pattern of BST thin film. The inset is the perovskite structure with Ti atom (silver) in center, O atom (red) at face, and Ba/Sr atoms (green) at corner. (**b**) EDX spectroscopy of BST films. The inset indicates the atomic percentage of each element.

Figure 1a shows a typical XRD pattern of perovskite-structural ferroelectric thin film, without the presence of any intermediate phases such as pyrochlore phase [17]. Since BST materials have a tetragonal P4mm symmetry, its lattice parameter *a* and *c* can be derived from XRD results (*a* = 3.9587 Å and *c* = 4.0313 Å), which is slightly larger than that of BST epitaxially grown on single crystal MgO substrate by molecular chemical vapor deposition (*a* = 3.9100 Å, *c* = 4.0030 Å) and very close to that of poly-crystalline BST on Pt by sputtering (*a* = 3.9870 Å, *c* = 4.0030 Å) [18,19]. It is mainly attributed to the thermal expansion mismatch of the substrates with Si. It is reported that the thermal expansion coefficient of Si is approximately 2.6 × 10^−6^ °C^−1^, which is far less than 6 × 10^−6^ °C^−1^ for BST and 12.6 × 10^−6^ °C^−1^ for MgO [19,20,21]. The other factor could be the lattice mismatch between the film and the Si substrate (*a* = 5.4300 Å). Energy dispersive X-ray spectroscopy (EDX) analysis, as shown in Figure 1b, detected four elements: Ba, Sr, Ti, and O, indicating the presence of BST thin films. More importantly, the percentage of Ti and O is 61.02:21.04 while that of Ba and Sr is 12.41:5.53 (The inset table), which is consistent with 3:1 for Ti/O and 7:3 for Ba/Sr in raw materials. The accurate and stable stoichiometric proportion in these samples is desirable for ferroelectric applications.

**Figure 2:**
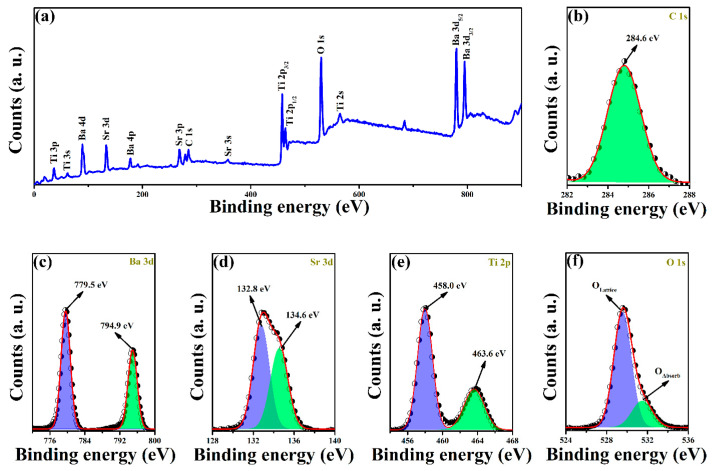
(**a**) Survey scanning of BST films. (**b**–**f**) High resolution XPS of C 1s, Ba 3d, Sr 3d, Ti 2p, and O 1s.

The X-ray photoelectron spectroscopy (XPS) technique is further performed to identify the elements and chemical states of the as-synthesized films. Figure 2a shows a XPS survey of BST thin film, which records C, Ba, Sr, Ti, and O. Subsequently, the high-resolution XPS spectra of C 1s, Ba 3d, Sr 3d, Ti 2p, and O 1s peaks in BST thin film are shown in Figure 2b–f. The C 1s at 284.6 eV can be used as a reference for the systematic shifts of the spectra (Figure 2b) [22]. Gaussian fitting analysis reveals that Ba 3d can be fitted by two peaks at 779.5 eV for Ba 3d_3/2_ and 794.9 eV for Ba 3d_5/2_, which are assigned to Ba atoms in the BST perovskite phase [23]. Similarly, two fitting peaks can be found in the spectra of both Sr 3d and Ti 2p. The binding energies of the former are 132.8 eV and 134.6 eV, while 458.0 eV and 463.6 eV in the latter. These results further confirm that Sr and Ti atoms relate to Sr^2+^ and Ti^4+^ in perovskites [24,25]. Furthermore, O 1s produces two peaks as shown in Figure 2f. The binding energy of 529.8 eV is corresponding to the binding energy of O atoms in the perovskite phase. Another peak at 531.6 eV is ascribed to the non-lattice oxygen due to the surface absorption [26]. Thus, XPS analysis confirms the formation of the perovskite phase in the view of element chemical bonding, which is consistent with the results of XRD and SEM results.

**Figure 3:**
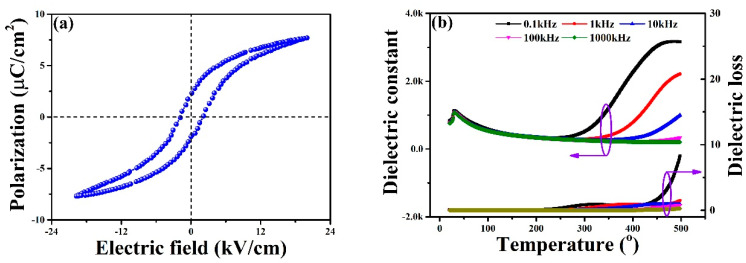
(**a**) Ferroelectric hysteresis loop of BST thin films. (**b**) Temperature-dependent dielectric constant and loss of the BST under diverse frequencies.

The ferroelectricity of BST thin films is demonstrated by the polarization-electric field (*P*-*E*) hysteresis loop. Figure 3a exhibits an asymmetric and slim *P*-*E* curve with the remnant polarization (*P*_r_) of 2.4 µC/cm^2^, compared to the values of 2.5 µC/cm^2^ for BST by pulsed laser deposition and 2.98 µC/cm^2^ for Zr-doped BST by sol-gel method [27,28]. Figure 3b shows the dielectric constant (*ε*_r_) and the loss (tan δ) versus temperature trend at a diverse frequency (0.1 kHz−1000 kHz). Notably, the typical ferroelectric materials follow the Curie-Weiss law above Curie temperature, which is expressed by: 1/*ε* = (*T* − *T*_c_)/C, where *T*_c_ is the Curie temperature and C is the constant. Therefore, a ferro-to-paraelectric (tetragonal-to-cubic) phase transition occurs at 31.2 °C, corresponded to the Curie temperature of BST. As the temperature ramps up, the *ε*_r_ initially decreases and is followed by rapid increasing. Meanwhile, the corresponding dielectric loss remains largely constant below 400 °C and then increases sharply at high temperatures. Similar phenomenon was also observed in BST bulk ceramics and (Pb,La)(Zr,Ti)O_3_ relaxor [29,30].

**Figure 4:**
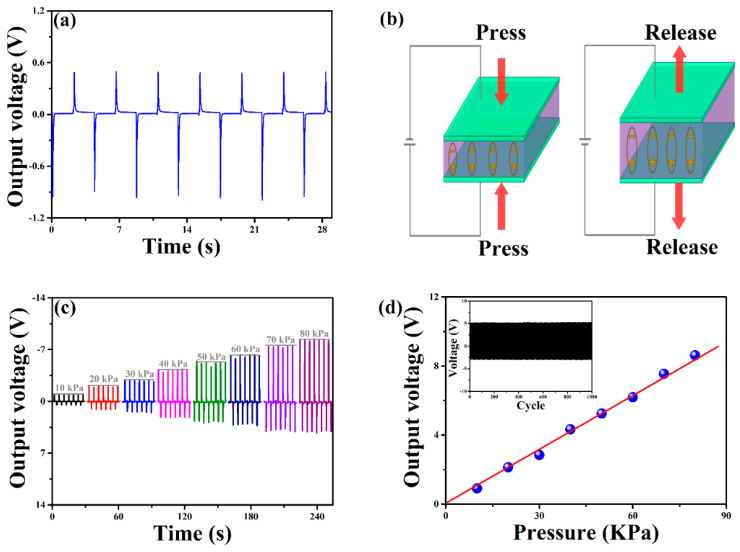
(**a**) Piezoelectric output voltage on BST at the applied periodic pressure of 10 kPa. (**b**) Schematic illustration of the piezoelectric effect of sandwiched BST between two electrodes. (**c**) Stress-triggered output voltage in the range of 10 kPa and 80 kPa. (**d**) Linear relationship between the performed pressure and the output voltage. The inset indicates the long-term operation stability of this device under the applied pressure of 50 kPa.

Mechanical manipulation in BST thin film is realized through a customer-designed testing system. The details on experimental sections can be found in our previous work [3]. Figure 4a shows the piezoelectric voltage response induced by applying a periodic mechanical pressure of 10 kPa. Notably, the output voltage demonstrates the similar periodicity, with a reliable maximum value of ~0.9 V. This can be mainly attributed to the piezoelectric effect of BST. As illustrated in Figure 4b, during the compression of the BST which is sandwiched between two electrodes, electrons are injected from the top electrode to the BST surface. During the subsequent releasing stage, charges transfer to the bottom electrode. Thus, the structure established the voltage potential between two electrodes, allowing electrons to flow through the top electrode to the bottom electrode, maintaining the electrostatic equilibrium. Generally, the relationship between the output voltage (*V*) and the applied pressure (*P*) is given by [31]:

*V* = (*hd*_33_*P*)/(*ε*_0_*ε*_r_) (1)

where *h*, *d*_33_, *ε*_0_, *ε*_r_ refers to the film thickness, the piezoelectric constant of BST, the permittivity of free space, and the relative dielectric constant of BST, respectively. As the mechanical pressure varies from 10 kPa to 80 kPa, a varying output voltage is generated, which is plotted in Figure 4c. In this case, the measured output voltages of BST thin film increase linearly in a range of 0.9 V to 8.6 V, which is in agreement with Equation (1) (Figure 4d). The sensitivity (*δ*) of the BST-based pressure sensors is defined as: *δ* = *V*/*P*, where *V* and *P* are the output voltage and the applied pressure. In the regime from 10 kPa to 80 kPa, the average sensitivity *δ* is calculated to be 0.10 mV·Pa^−1^, which is considerably higher than the 0.05–0.07 mV·Pa^−1^ range achieved by other ferroelectric sensors [32,33]. More importantly, almost no degradation in performance was observed, even after 1000 cycles (the inset of Figure 4d), indicating excellent operation stability of our BST-based sensing device.

**Figure 5:**
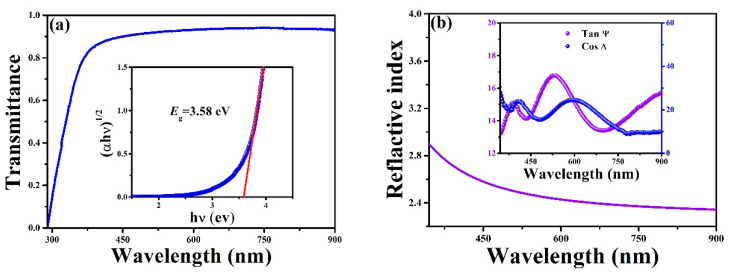
(**a**) Transmittance curve of the resulted BST. The inset reveals the corresponding calculated band gap. (**b**) Refractive index of BST as a function of wavelength. The inset is the Tan Ψ and Cos Δ from the ellipsometry measurement.

Figure 5a displays the typical transmittance curve of the BST film measured with a UV-Vis Spectrometer, indicating a high transparency under visible to near-infrared light. The inset is a plot of the absorption edge, which can be used to derive the optical band gap of BST using: (*αhυ*)^2^ = C(*hυ* − *E*_g_), where *α*, *hυ*, and *E*_g_ are the absorption coefficient, the photon energy, and the optical band gap, respectively. From the inset, the optical band gap of BST is estimated to be 3.58 eV, which is consistent with the data reported in the literature [34,35]. The measured ellipsometric parameters Tan Ψ and Cos Δ were applied, respectively, to derive the refractive index (*n*) of BST films (Inset of Figure 5b). In this case, the *n* versus the wavelength (*λ*) can be expressed as: *n*(*λ*) = A + (B/*λ*^2^) + (C/*λ*^4^), where A, B, and C are the Cauchy parameters [16]. The as-grown BST sample exhibits a refractive index of 2.42 at the wavelength of 632 nm (Figure 5b). This value is comparable to 2.43 for Pb(Zr,Ti)O_3_ and 2.34 for (Pb,La)(Zr,Ti)O_3_ and slightly smaller than that of Pb(Mg_2/3_Nb_1/3_)O_3_-PbTiO_3_ (PMN-PT, *n* = 2.599) [36,37,38]. The refractive index in perovskites can be easily manipulated by external stimulus such as the electric field, e.g., birefringence, which in turn provides the opportunity to manipulate the optical performances of BST [39].

**Figure 6:**
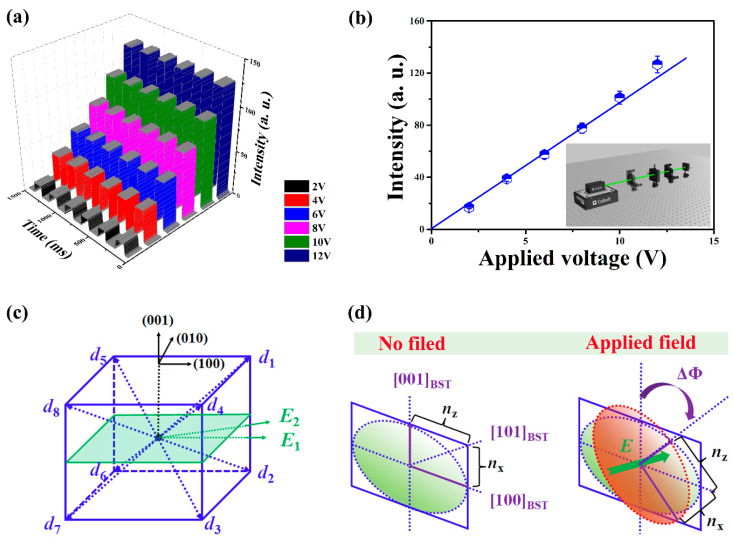
(**a**) Light manipulation as functions of the applied voltage. (**b**) The relationship between the laser intensity and the applied voltage. The inset is the schematics of experimental setup. (**c**) Eight possible spontaneous polarization directions in BST along (001). (**d**) reveals the projected ellipses on (001) plane of BST with and without the applied electric field. Applying an electric field *E* change the birefringence (Δ*n*) and rotates the optical axis as well.

In addition to mechanical sensitivity, the optical manipulation in BST films can be also realized. Figure 6a demonstrates the laser intensity modulation under diverse applied voltages. Notably, the relationship between the intensity and the applied voltage is linear, evidenced by Figure 6b. Utilizing a modified Senarmont compensator style, the inset of Figure 6b is the schematics of experimental setup, equipped with a stabilized He–Ne laser, a polarizer, an analyzer, and a photodetector [40]. The output intensity (*I*), recorded by the photodetector, can be given by: *I* = 1/2 *I*_0_(1 + sinΓ) = *I*_0_(1 + sin(2π*l*Δ*n*/*λ*)), where *I*_0_ is the initial intensity of the laser, Γ is the phase retardation, *l* is the thickness of BST, and Δ*n* is the birefringence variation under the applied electric field. As we know, the electric field *E* is applied perpendicular to the ferroelectric film, leading to a shift in the refractive index. Thus, the field dependence *E* of the index *n*(*E*) is given by [39]:

*n*(*E*) = *n* − 1/2 *rn*^3^*E* + 1/2 *ξn*^2^*E*^2^ (2)

where *r* and *ξ* are the Pockels and Kerr effect coefficients, respectively. Tetragonal BST is a positive uniaxial optical crystal with its optical axis along the polarization direction. When applying an *E*_1_ or *E*_2_ electric field along the (110) direction, for example, using the coordinate system shown in Figure 6c, the spontaneous polarization inside the (110) plane was poled along the O_d1_ and O_d2_ directions. Figure 6d displays the refractive index ellipse in BST, showing the refractive index *n*_x_ and *n*_z_ along different crystalline directions (left). Applying an electric field *E* changes the birefringence (Δ*n*) and rotates the optical axis depending on the orientation of *E*. As for birefringent materials such as BST, *n*_x_ = *n*_y_ = *n*_o_ and *n*_z_ = *n*_e_, where *n*_o_ and *n*_e_ are the ordinary and extraordinary refractive indices of the medium, respectively [41]. Therefore, the modulation simplifies in Equation (2) to *n*_o_(*E*) = *n*_o_ − 1/2 *r*_13_*n*_o_^3^*E* or *n*_e_(*E*) = *n*_e_ − 1/2 *r*_13_*n*_e_^3^*E*. When defining an effective electro-optic coefficient as *r*_eff_ = *r*_33_ − *r*_13_(*n*_o_^3^/*n*_e_^3^), the birefringence (Δ*n*) can be given by: Δ*n* = *n*_e_ − *n*_o_ = 1/2 *r*_eff_*n*^3^*E*_._ Generally, the change in refractive index induced by the electric field is very small for direct measurements. Therefore, the change in refractive index is always replaced by an optical path difference or phase change in practical experiments [39]. Herein, the electro-optic coefficient *r*_eff_ of BST films is estimated to be 83.5 pmV^−1^. It is noteworthy that this value is also comparable with the commonly used transverse BST coefficient in (111)-oriented BST (87.8 pmV^−1^) and is far superior to that of LiNbO_3_ crystals (31.8 pmV^−1^), opening up new avenues to replace LiNbO_3_ for enabling future high-speed and cost-effective optical communication networks [42,43].

## 4. Conclusions

To sum, barium strontium titanate thin films were directly deposited on silicon substrates by sputtering technique. Their crystal phase, spontaneous polarization and temperature-dependent dielectric characteristics were subsequently studied. In addition to the high transmittance over a wide wavelength regime, the as-grown BST films have exhibited a remnant polarization of 2.4 μC/cm^2^, a ferro-to-paraelectric (tetragonal-to-cubic) phase transition temperature of 31.2 °C, and a broad optical bandgap of 3.58 eV. Owing to their excellent ferroelectric performances, the resulted BST films exhibit a highly sensitive pressure sensor with an average sensitivity of 0.10 mV·Pa^−1^ in the 10–80 kPa regime and excellent operation stability of 1000 cycles or above. Additionally, they can be also used to manipulate optical properties simply by applying a smaller voltage, which is linked to the electric field-induced Pockels effect. An electro-optic coefficient of 83.5 pmV^−1^ is observed. This study paves the way for a new opportunity for on-chip integrations with various electronics into sensors via CMOS-comparable technique, allowing for low power consumption and multimodel functionalities.

## Data Availability

The data presented in this study are available on request from the corresponding author.
